# Exploring the Effect of Specimen Size on Elastic Properties of Fused-Filament-Fabrication-Printed Polycarbonate and Thermoplastic Polyurethane

**DOI:** 10.3390/ma17112677

**Published:** 2024-06-01

**Authors:** Charul Chadha, Gabriel Olaivar, Mahmoud A. Mahrous, Albert E. Patterson, Iwona Jasiuk

**Affiliations:** 1 Department of Mechanical Science and Engineering, University of Illinois at Urbana-Champaign, Urbana, IL 61801, USA; 2 Department of Industrial and Enterprise Systems Engineering, University of Illinois at Urbana-Champaign, Urbana, IL 61801, USA; olaivar2@illinois.edu; 3 Department of Civil and Environmental Engineering, University of Illinois at Urbana-Champaign, Urbana, IL 61801, USA; mahrous2@illinois.edu; 4 Faculty of Manufacturing and Mechanical Engineering Technology, Department of Engineering Technology and Industrial Distribution, Texas A&M University, College Station, TX 77843, USA; aepatterson5@exchange.tamu.edu; 5 J. Mike Walker ’66 Department of Mechanical Engineering, Texas A&M University, College Station, TX 77843, USA

**Keywords:** size effects, additive manufacturing, fused filament fabrication, architected materials, design for additive manufacturing

## Abstract

Additive manufacturing (AM) is often used to create designs inspired by topology optimization and biological structures, yielding unique cross-sectional geometries spanning across scales. However, manufacturing defects intrinsic to AM can affect material properties, limiting the applicability of a uniform material model across diverse cross-sections. To examine this phenomenon, this paper explores the influence of specimen size and layer height on the compressive modulus of polycarbonate (PC) and thermoplastic polyurethane (TPU) specimens fabricated using fused filament fabrication (FFF). Micro-computed tomography imaging and compression testing were conducted on the printed samples. The results indicate that while variations in the modulus were statistically significant due to both layer height and size of the specimen in TPU, variations in PC were only statistically significant due to layer height. The highest elastic modulus was observed at a 0.2 mm layer height for both materials across different sizes. These findings offer valuable insights into design components for FFF, emphasizing the importance of considering mechanical property variations due to feature size, especially in TPU. Furthermore, locations with a higher probability of failure are recommended to be printed closer to the print bed, especially for TPU, because of the lower void volume fraction observed near the heated print bed.

## 1. Introduction

Additive manufacturing (AM) has gained widespread adoption due to its ability to produce intricate geometries, customize designs, and improve part integration. This versatility enables the creation of components with diverse geometries, dimensions, and hierarchical structures [1,2,3]. However, predicting the response of these components under various loading conditions remains challenging, as material properties can vary due to the change in the cross-sections of the specimens. Experiments aimed at developing material models are usually guided by testing standards, usually with smaller sample cross-sections. As a result, uncertainties arise in scaling material properties across diverse cross-sections. This uncertainty stems from a dearth of research investigating the impact of size on the properties of 3D-printed materials.

Prior studies have highlighted the influence of the thickness of the specimen on material properties in metals [4,5,6,7,8]. Tensile tests on 316L stainless steel and Ti–6Al–4V samples manufactured using laser powder bed fusion revealed that yield strength, ultimate tensile strength, and Young’s modulus reduce with a decrease in thickness of the tensile samples [4,5]. These decreases in material properties were attributed to the increased void volume fraction and surface roughness [5]. A study conducted by He et al. [4] demonstrated that employing a standard material model across various cross-sections during simulations can lead to discrepancies between computed and experimental results. As a result, variations in material properties due to size of the specimen need to be understood and taken into account during the design stage.

In the context of polymeric materials, the effect of size has been studied for specimens manufactured using fused filament fabrication (FFF), also referred to as fused deposition modeling (FDM, trade name owned by Stratasys Inc., Eden Prairie, MN, USA [9]). FFF is one of the most widely used AM techniques. Developed initially as a tool for rapid prototyping, FFF has emerged as a pivotal manufacturing process in several industries (automotive [10], aviation [11], medicine [12], and construction [13]) due to advantages like ease of material handling, low cost, and portability. The strength of the materials printed using FFF, however, was found to reduce with an increase in size of the specimen [14,15,16,17]. Wu et al. [16] explained that a significant plastic zone could be observed in smaller specimens, enabling them to absorb relatively higher fracture energy, resulting in greater strength. The mechanism was observed to follow well-known size effect laws (Bazant’s theory [18], modified Bazant’s theory [19], and Carpinteri’s theory [20]). Traditionally, size effects are defined as the effect of the characteristic size of a structure on its nominal strength when geometrically similar structures are compared. Nurizada and Kirane [21] also studied variation in fracture toughness due to size effects along three different orientations. The researchers acknowledged that high pore density typically observed at the top of the specimen can influence the size effect. However, they rejected this assumption because the initial elastic slope for the samples under study did not change appreciably with size. Similar conclusions were drawn by Sadaghian et al. [15], where the modulus of the sample was not found to be affected by its size. These results contradict Guessasma et al. [22], who observed a slight difference in response in the elastic region under compression for the ABS. However, a clear trend was not reported. Size effects have also been reported for other manufacturing processes, like polymers manufactured using PolyJet [21] and ceramics manufactured using binder jetting [16].

**Figure 1:**
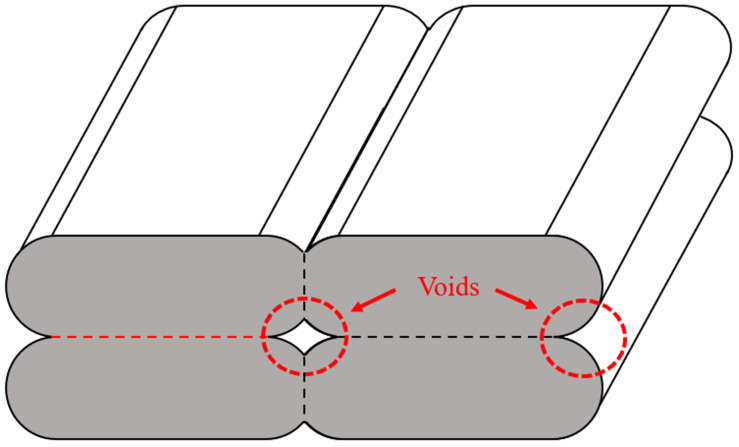
Mesostructure formed during FFF.

Typically, the materials produced using FFF exhibit lower material properties compared to traditional manufacturing processes like injection molding. This reduction in material properties is primarily due to voids generated during the FFF process. Techniques like scanning electron microscopy (SEM) and micro-computed tomography (micro-CT) have been used to study the voids formed during FFF. While SEM is a valuable tool in analyzing the cross-section of the sample, this technique primarily captures two-dimensional (2D) images of the cross-section with a limited depth. Three-dimensional (3D) images of the samples, on the other hand, can be captured using micro-CT. Guessasma et al. [23] were among the first to employ micro-CT to examine the internal structure in FFF. They found that voids were formed in the specimens due to (a) incomplete polymer diffusion between the deposited material (beads or raster), (b) abrupt changes in print direction, and (c) lack of fusion between the layers. The voids formed due to incomplete diffusion were found to be long, continuous defects aligned along the direction of bead deposition. These voids were found to repeat periodically, resulting in the formation of a mesostructure (Figure 1), which affects the mechanical properties of the printed material [24,25]. Minor variations in the cross-section and oscillations were observed when focused on single voids due to irregularities such as ghosting and rippling [26].

The geometry of the mesostructure is influenced by a range of printing parameters, such as bead width [27,28], layer height [29,30,31,32,33], print speed [10,34], and print temperature [35,36], leading to variations in mechanical properties. The factors affecting the formation of voids can be divided into two categories: factors affecting thermal history (such as print speed, print temperature, and bed temperature) and geometrical factors like bead width and layer height [37].

Efforts have been made to co-relate the effect of voids on the effective material properties using analytical solutions and finite element analysis (FEA) [27,33,34,38,39,40,41,42]. Additionally, solutions using fast Fourier transform (FFT) [26] and semi-analytical techniques [43] have been developed to overcome shortcomings of purely analytical and FEA-based solutions (like mesh dependency and a large computational time). These studies have shown that voids reduce the elastic modulus of the printed material and act as sources of failure. Larger voids (resulting from parameters such as a larger layer height) also increase the anisotropy of the material.

However, conflicting conclusions about the influence of layer height on the porosity distribution have been reported in the literature. In one study, increasing layer height has been shown to reduce the number and size of the pores at the layer interfaces [29]. In another study, increasing layer height increased the number of pores and size of voids [31,32].

The examination of prior research highlights a pressing need for additional exploration of the influence of specimen size on the mechanical properties of 3D-printed polymers. This research gap not only impedes innovation but also restricts the practical application of AM, in particular, FFF, in the large-scale production of polymer-based components. Thus, there is a compelling impetus to examine variations in material properties due to size across different types of polymers printed using FFF. Undertaking such a study would not only substantially enrich the current body of knowledge but also help design engineers to predict the response of a component without the need for extensive testing, thereby expediting the creation of more efficient and effective large-scale AM products and conserving resources.

Thus, in this paper, we investigate the compressive response of the specimens printed by FFF at various sizes. We consider two materials, a stiff polycarbonate (PC) and soft thermoplastic polyurethane (TPU), to gain insights into the effect of the material choice. Additionally, given that the properties of printed materials are influenced by void geometry, we also varied layer height to explore the relationship between void volume fraction and size of the specimen. This study provides new insights into the effects of specimen size and layer height in FFF-printed materials and provides a framework for more comprehensive future investigations.

This paper has five sections. Section 2 delves into the specifics of material selection, sample preparation, and experimental methods employed. Section 3 presents the findings. A discussion of these results is in Section 4, followed by the conclusions, summary, and suggestions for future work in Section 5.

## 2. Materials and Methods

### 2.1. Sample Preparation

Cubic specimens with side dimensions of 8 mm, 12 mm, and 20 mm were 3D printed using the Sovol SV02 (Sovol3D, Shenzhen, China) at the layer height of 0.1 mm, 0.2 mm, and 0.4 mm. The specimens were printed based on the full factorial design of experiments (FF-DOE). To construct FF-DOE, three levels were considered for the size of the specimens (8 × 8 × 8 mm^3^, 12 × 12 × 12 mm^3^, and 20 × 20 × 20 mm^3^) and layer height (0.1 mm, 0.2 mm, and 0.4 mm). This experimental design resulted in a DOE with a 3 × 3 number of cases, as shown in Table 1.

Two polymers, polycarbonate (PC) and thermoplastic polyurethane (TPU), were chosen for this study because of their contrasting mechanical properties. PC has a high modulus and is an amorphous material, whereas TPU is a relatively softer and semi-crystalline material [44,45,46]. To ensure consistent printing conditions, the nozzle size, layer width, air gap, raster angle, print orientation, wall count, print speed, and nozzle/bed temperatures were held constant across all the prints. The specimens made from PC were printed using a 0.6 mm nozzle size, nozzle temperature of 260 °C, print speed of 40 mm/s, and bed temperature of 110 °C, while the specimens made from TPU were printed at a nozzle temperature of 230 °C, print speed of 15 mm/s, and bed temperature of 70 °C. The bead width and raster angles were kept constant at 0.6 mm and +45°/−45°, respectively. Four specimens for each DOE point shown in Table 1 were tested for both materials. Thus, in total, 4 × 2 × 3 × 3 (number of specimens× (two levels corresponding to two materials) × (three levels corresponding to the three cube dimensions) × (three levels corresponding to three sample layer heights)) specimens were tested. The weight and dimensions of each specimen were recorded before testing to calculate the equivalent density (mass/volume).

### 2.2. Micro-CT Imaging

Micro-CT is a nondestructive imaging method that employs X-rays to generate two-dimensional (2D) trans-axial projections of specimens and subsequently reconstructs them into a comprehensive 3D representation using specialized reconstruction software. A grayscale value is assigned to each spatial position, representing the local phase density. In this study, PC and TPU specimens were scanned using a Rigaku CT Lab HX 130 micro-CT machine (Rigaku, Tokyo, Japan). The specimens were placed on a rotating platform situated between the X-ray source and detector screen. The voltage, current, and distance from the source were 130 kV, 60 μA, and 20 mm, respectively. A total of 1930 2D images were captured in continuous acquisition mode over a period of 68 min, resulting in a resolution of 5.3 μm.

**Figure 2:**
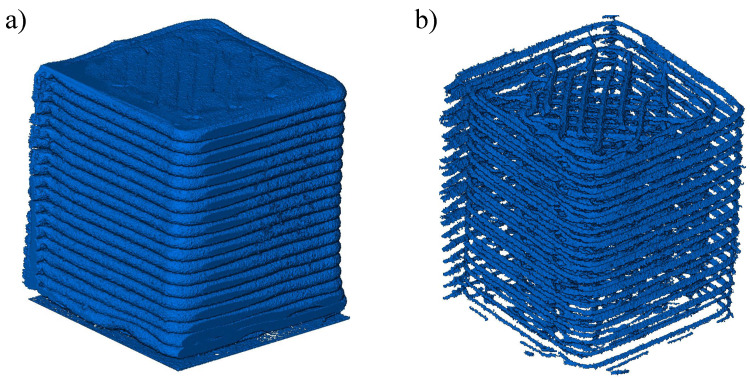
Images obtained after (**a**) thresholding and (**b**) inverting the thresholding to view voids.

The micro-CT scans were converted into TIF images and reconstructed using Amira 3D 2022.2 software. The gray-level images were then converted into binary images using interactive thresholding to separate the pores from solid features. The threshold values were adjusted twice: first, to visualize the deposited material, and then to render a 3D image of the voids. Thresholding was followed by eliminating artifacts of voxel size less than 20 units using the “remove small spots option”. Figure 2 shows the images obtained after the two thresholding steps for a PC specimen. The segmentation process in the Amira software is described in detail by Mahrous et al. [47]. The specimens were also cropped to study the deposition of beads according to the raster angle, which allowed for the identification of repeating architecture at the mesoscale. Label analysis was then conducted to determine the void volume and connectivity. The void volume fraction was calculated using Equation (1).

(1) $$V_{f}=\frac{\mathrm{Volume}\;\mathrm{of}\;\mathrm{voids}}{\mathrm{Total}\;\mathrm{volume}\;\mathrm{of}\;\mathrm{the}\;\mathrm{specimen}}$$

### 2.3. Compression Testing

**Figure 3:**
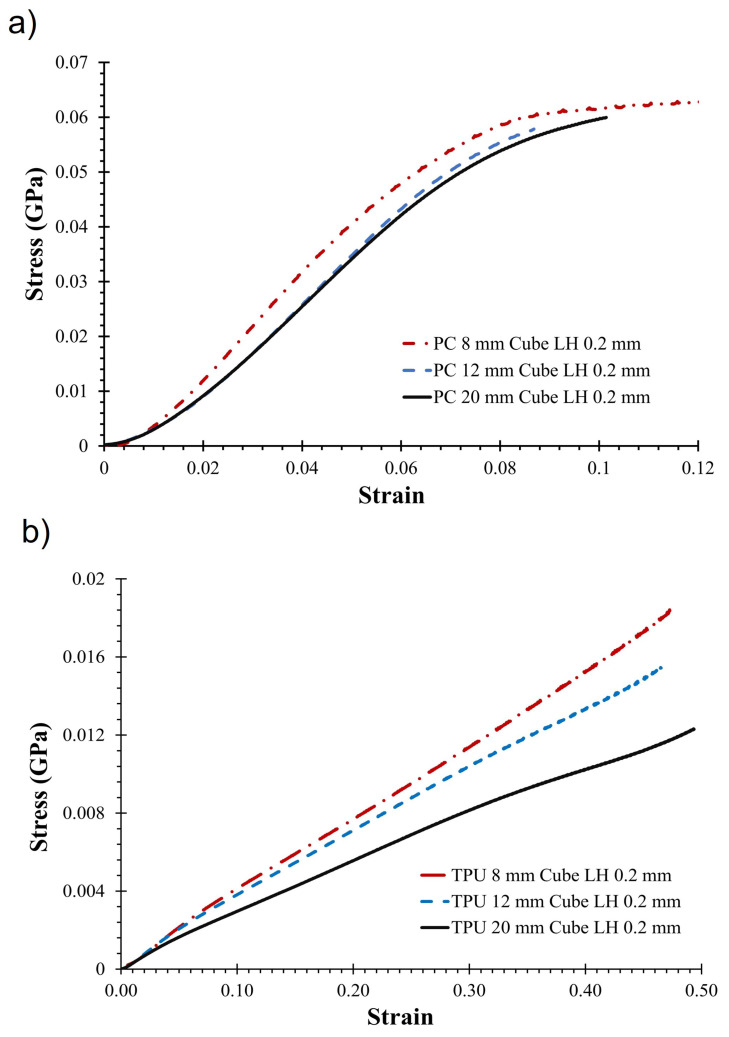
Stress–strain curve for (**a**) PC sample and (**b**) TPU printed with 0.2 mm layer height.

The cubic specimens were subjected to compression along the print direction using an MTS Alliance RT/30 load frame (MTS Systems, Eden Prairie, MN, USA). The specimens were compressed at a rate of 0.1 mm/min following the ASTM D695-15 standard [48]. Cubic samples were used instead of cuboid samples, as recommended by the standard, to prevent buckling. Load cells with capacities of 30 kN and 10 kN were used to obtain the load–displacement data. The stress–strain curves were then obtained from the collected data. Figure 3 shows the stress–strain curves for TPU and PC specimens printed at different sizes using 0.2 mm layer height.

## 3. Results

### 3.1. Micro-CT Image Analysis

**Figure 4:**
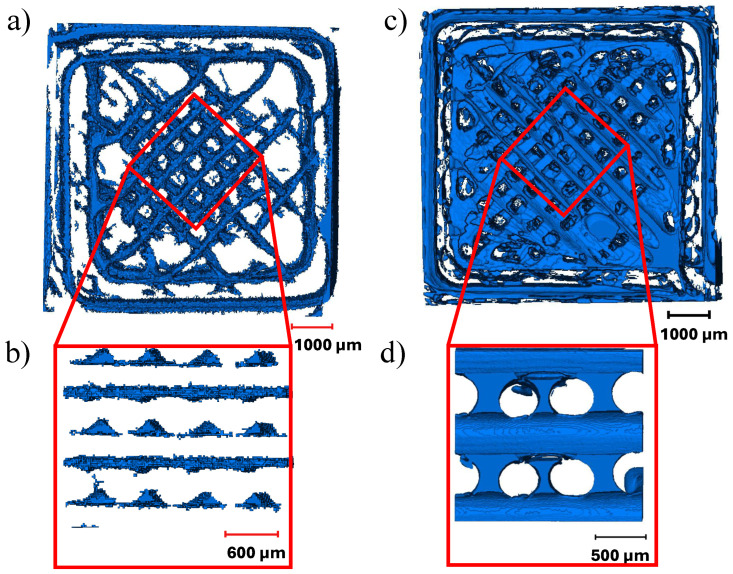
(**a**) Top view of the voids formed in 8 mm × 8 mm × 8 mm PC specimen printed at 0.4 mm layer height, (**b**) side view of voids formed due to insufficient polymer diffusion; (**c**) top view of the voids formed in 8 mm × 8 mm × 8 mm TPU specimen printed at 0.4 mm layer height, (**d**) side view of voids formed due to insufficient polymer diffusion.

Micro-CT images were studied to identify variations in the mesostructure and voids formed during FFF. Three types of defects were observed: voids caused by abrupt changes in the printing directions, insufficient polymer diffusion between deposited beads, and a lack of fusion between layers similar to the ones reported by Guessma et al. [23]. It is interesting to note that a lack of fusion between layers was rarely observed in PC but was noticed more frequently in the specimens printed from TPU. Voids formed due to abrupt changes in print direction tend to stack across different layer heights. Eiliat and Urbanic [49] also noted the stacking of voids resulting from changes in printing direction, describing them as having a ’chimney’-like structure. The defects are shown in Figure 4.

Of the three types of defects, the majority of voids were formed due to insufficient diffusion between the beads. These voids were observed between the two deposited beads along the raster angle. As described by Paux et al. [26], the voids formed due to insufficient diffusion had a consistent cross-section along the raster angle and were observed to be repeating periodically within the layer and between the layers. However, we noticed that while the dimensions of the void cross-sections were consistent within a layer, the dimensions varied along the print direction (between the layers). Smaller voids were typically found near the print bed, whereas voids with larger cross-sections were observed at the top of the specimens.

Furthermore, while the voids resulting from inadequate diffusion were not interconnected in the PC, they formed a connected network in TPU. Significant air gaps between deposited beads have been reported in the literature [50]; nevertheless, the interconnectivity between the voids in TPU has not been discussed previously. Kasmi et al. [51] reported void volume fractions between the range of 16 and 35% for a 100% flow rate. High void volume fractions are observed because the bead width of the actual deposited material is less than the width specified in the slicer software (Cura 4.8.0). Lin et al. [50] described this behavior as a “hungry feeding” mechanism caused by low glass transition temperature $T_{g}$. The $T_{g}$ of TPU is often less than room temperature, resulting in a low Young’s modulus and higher flexibility at room temperature. Due to increased flexibility, when the TPU is extruded from the nozzle, it initially squeezes and then stretches along the raster angle, decreasing the bead thickness. Extrusion die swelling, necking, and shrinkage behaviors may have an impact on the dimensional accuracy of the deposited bead [52,53]. As a result, the printer must be carefully calibrated to compensate for this effect.

The volume fraction of the voids observed in the specimens made from PC was between 6% and 8%. In contrast, the void volume fractions of the specimens made from TPU were significantly higher, ranging from 26% to 34%. It should be noted that providing an exact void volume fraction was challenging because the value depends on the threshold value selected for the micro-CT images. Therefore, a range is provided.

**Figure 5:**
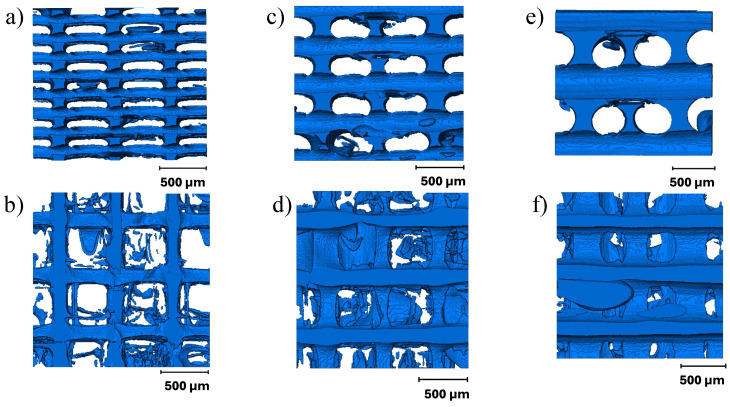
Variation in the distribution of voids due to varying layer height: (**a**,**b**) side and top view of voids formed in 8 mm × 8 mm × 8 mm specimens printed at 0.1 mm layer height, (**c**,**d**) side and top view of voids formed in 8 mm × 8 mm × 8 mm specimens printed at 0.2 mm layer height, (**e**,**f**) side and top view of voids formed in 8 mm × 8 mm × 8 mm specimens printed at 0.4 mm layer height.

Smaller sections of the specimens were cropped to study the effect of layer height on the void size and connectivity. A lower layer height resulted in voids with smaller cross-sections (Figure 5). A reduction in void cross-section due to the decrease in layer height has been reported previously [33]. However, the frequency of voids along the printing direction increased because additional layers were required to print the same height. Figure 5 compares the formation of voids across different layer heights in the PC and TPU samples. As a result, the smallest void volume fraction was observed in specimens printed with a 0.2 mm layer height compared to 0.1 mm and 0.4 mm.

### 3.2. Modulus and Specific Energy Absorption

**Figure 6:**
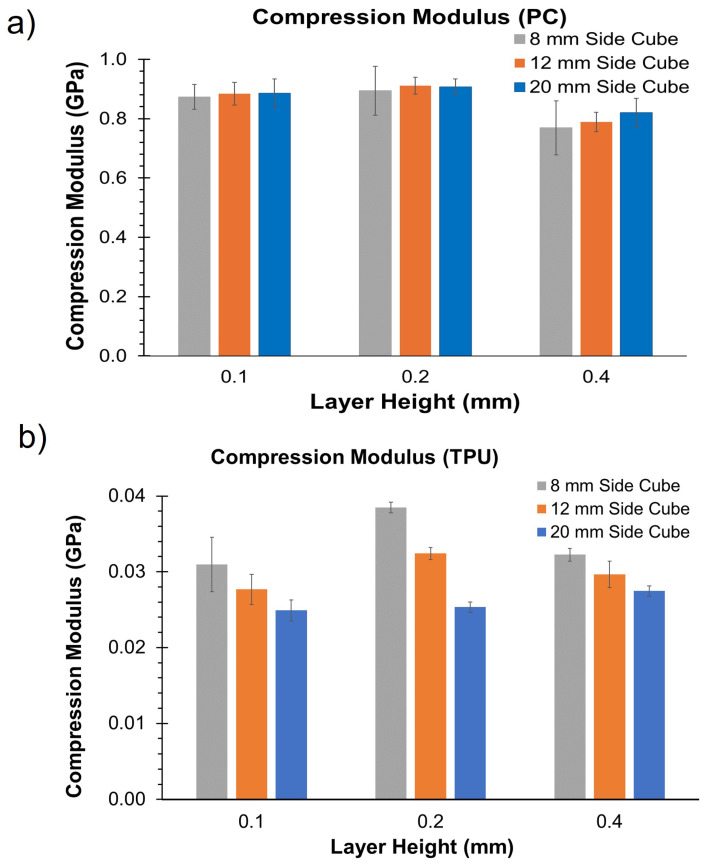
Compression moduli of (**a**) PC and (**b**) TPU.

The compression modulus was calculated for the cubic specimens printed from PC and TPU along the print direction from the linear region of the stress–strain curve. The average compression modulus and corresponding standard deviation for each point in DOE are given in Appendix A. Figure 6 compares the compression modulus for different layer heights and specimens of different sizes. We observe that the mean compression modulus did not vary significantly with the size of the specimen for the 0.1 mm and 0.2 mm layer height in the case of PC, but it did vary for the 0.4 mm layer height. In contrast, the compression modulus decreased with an increase in the size of the specimen for TPU across all layer heights. This result is in contrast with observations from metal AM. In metal AM, the modulus of the specimens decreased with a reduction in the thickness. The contradictory relationship observed in TPU can be due to multiple reasons. Firstly, in metal AM, specimens with thin cross-sections (6.25 mm to 0.4 mm) were tested under tensile loading, where, due to the stretching, the effective load-resisting area decreased with an increase in load [5]. Secondly, the mesostructure and defects formed during powder bed fusion are different from the ones formed in FFF.

No significant variation in modulus due to changes in the size for PC is consistent with the observations made by Sadaghai et al. [15]. On the other hand, the observations for TPU appear to contradict this result and align with the findings of Wu et al. [16], who reported a reduction in modulus when the size of the specimens increased for plasters fabricated using the binder jetting process. Highest compression modulus was observed at the layer height of 0.2 mm for both PC and TPU, across different specimen dimensions.

**Figure 7:**
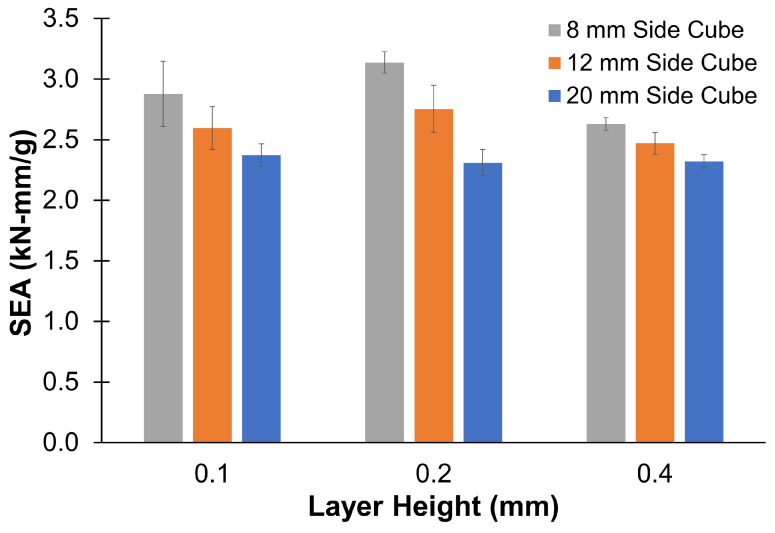
Specific energy absorption (SEA)—TPU.

When comparing the stress–strain curves, nonlinearity was observed at small strains across all samples. The initial nonlinearity region was smaller in the PC compared to the TPU. Because significant nonlinearity was observed in TPU, the specific energy absorption (SEA), as defined in Equation (2), was also calculated up to a 40% strain. Figure 7 compares the SEA for TPU. A similar trend was observed in SEA as in compression modulus.

(2) $$\mathrm{SEA}=\frac{\mathrm{Area}\;\mathrm{under}\;\mathrm{load-displacement}\;\mathrm{curve}}{\mathrm{Weight}\;\mathrm{of}\;\mathrm{the}\;\mathrm{specimen}}$$

## 4. Discussion

The significance of layer height and size of the specimens on compressive elastic moduli was further investigated using analysis of variance (ANOVA). ANOVA is a statistical analysis method used to determine whether the difference between means of two or more groups is significant. Minitab 21.4.3.0 was employed to perform the ANOVA. Before conducting the ANOVA, an Anderson–Darling test was administered to assess the normality of the data. The results of the test revealed that while the compression modulus for PC was normally distributed, the compression modulus of TPU was not. Consequently, a Box–Cox transformation was applied to perform the ANOVA for TPU. The results of the ANOVA for PC and TPU are presented in Table 2 and Table 3, respectively. The significance of the terms employed for the ANOVA analysis is explained in Appendix B. The *p*-values obtained from the ANOVA (Table 2 and Table 3) suggest that the layer height and specimen size significantly influence the elastic moduli of TPU samples. However, only layer height demonstrated a statistically significant impact on the elastic moduli of the PC.

**Figure 8:**
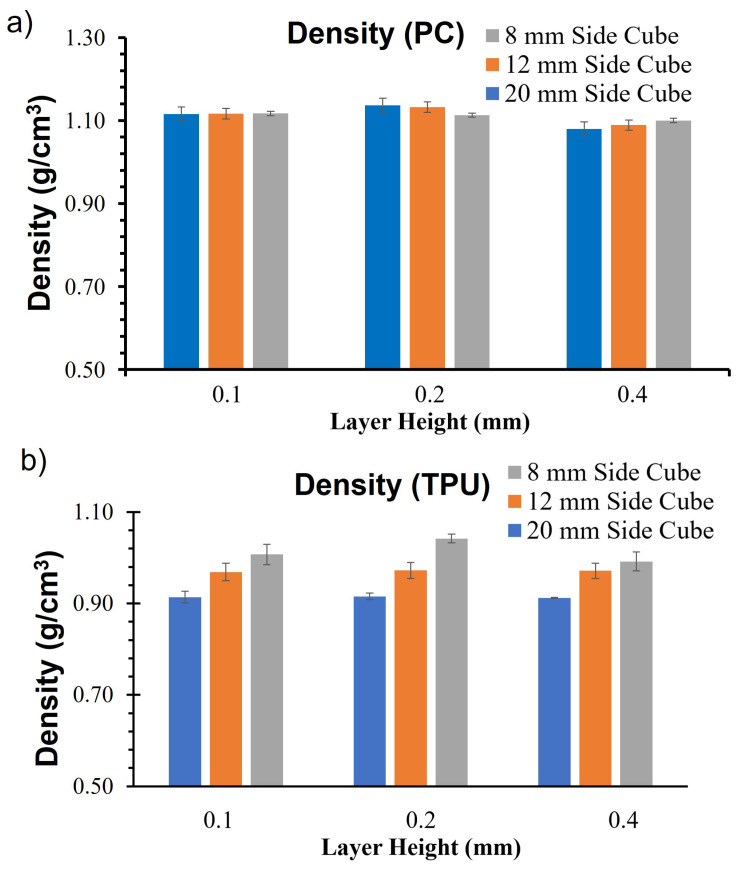
(**a**) Equivalent density PC and (**b**) equivalent densities TPU.

The specimen size affects the material properties in TPU due to its substantial void volume fraction and low elastic modulus. When subjected to compression, the deposited material initially deformed to fill the voids before transferring the loads, resulting in noticeable nonlinearity at low strains (less than a 10% strain for TPU). The higher moduli of the smaller TPU specimens can be attributed to several factors. As explained by Wu et al. [16], a large proportion of the surface experiences high strain in smaller specimens, whereas larger specimens only have a small area of high strain. Consequently, smaller specimens have a higher proportion of material engaged in load-carrying mechanisms, and the compressive strain is fully developed in these specimens. In addition, smaller TPU specimens had higher density (Figure 8). This increase in density can be attributed to the lower thermal gradient observed by deposited material because of (a) proximity to the heated bed and (b) less time required to print a layer.

The thermal gradient in FFF can be controlled using printing parameters like print speed, print temperature, and print bed temperature. A reduced print speed and higher bed temperature can reduce the thermal gradient, promoting polymer diffusion between the deposited beads [38,54,55,56,57].

**Figure 9:**
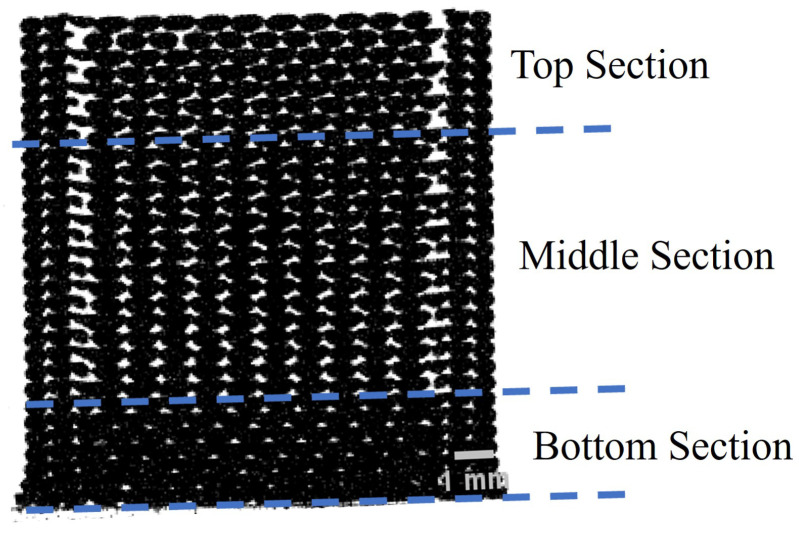
Three sections in TPU 12 mm × 12 mm × 12 mm sample printed at layer height of 0.4 mm.

Due to variations in the thermal gradient, a specimens printed using FFF can be divided into three distinct sections: the bottom, middle, and top (Figure 9). The distinction between the three sections is more prominent in taller specimens. The bottom section is closest to the print bed. The material in this section undergoes slow cooling rates due to the heated print bed, leading to higher polymer diffusion, leading to denser sections. In the middle section, the effect of the heat supplied by the print bed reduces due to a considerable distance from the heat source. The cooling rate in the middle section is determined by printing parameters such as print temperature, print speed, and ambient temperature. The beads deposited in this section experience repeated heating and cooling cycles due to multiple layers being added above them. The polymers deposited in the top section had a similar cooling rate. However, the material deposited in this section experienced fewer repeated heating and cooling cycles because of the limited amount of material deposited on top, resulting in a smaller wetted area and higher void fraction.

Additionally, a higher density in smaller specimens can also result from the shorter time needed to print each layer. Due to the smaller cross-section, the distance traveled by the printer’s nozzle is reduced. Consequently, the material from the preceding layer may still be at a higher temperature, leading to better diffusion. Further, studies are required to relate the thermal history of the material to the density of the printed specimens. The variation in densities among the three sizes is more pronounced in TPU than in PC (Figure 8). The highest compression modulus was observed at the layer height of 0.2 across all the cubes. However, a higher variation in modulus due to the size of the specimen was also observed at a 0.2 layer height in TPU.

One of the advantages of AM is its ability to print intricate components with varying geometries and dimensions based on the design requirements. However, the material properties of features designed inside a component can vary based on its size. Thus, design and analysis engineers should take variations in material properties due to changes in dimensions into account during the design process to predict the response of the component accurately.

The current study investigated the compression modulus variations along the print direction, focusing on the effect of specimen size. However, this study has several limitations that can be explored in the future. One limitation is that all specimens were printed on a heated print bed, leading to a higher density in smaller TPU specimens. Future studies could explore the underlying reasons for this density variation. Additionally, testing specimens printed farther away from the print bed, thereby reducing the thermal effects from the print bed, could provide a more comprehensive understanding of variation in material properties due to sample size. Further studies could also explore size effects on properties measured perpendicular to the print directions for various infill densities and different sets of printing parameters. As discussed earlier, thermal analysis co-relating the size of voids and material properties for different specimen geometries can also be beneficial for TPU.

## 5. Conclusions

Cubic specimens with varying dimensions and layer heights were printed using filaments made of PC and TPU. The internal structures and defects present in the specimens were analyzed using micro-CT. The observations from the study are as follows:

- The width of deposited beads in TPU decreases during the deposition process, resulting in the interconnected void network. The voids formed due to incomplete polymer diffusion in PC were aligned along the raster angle and did not connect across layers.
- Size of the specimen and layer height had a statistically significant impact on compression modulus for TPU, with smaller specimens exhibiting a higher modulus. However, only layer height had a statistically significant effect on the compression modulus of PC.
- The highest compression modulus was observed for a layer height of 0.2 mm across all specimens.
- Design and analysis engineers should account for the variation in elastic properties due to the size and density of the material during the design stage for TPU.
- The sections of the designed component that are expected to experience increased loads or have a higher likelihood of failure should ideally be printed closer to the print bed.

Further studies are required to test specimens of varying sizes printed farther away from the print bed to provide a more comprehensive understanding of the variations in material properties attributable to specimen size for TPU.

## Figures and Tables

**Table 1:** DOE used for the analysis.

SNo.	Cube Dimensions (mm^3^)	Layer Height (mm)
1	8 × 8 × 8	0.1
2	8 × 8 × 8	0.2
3	8 × 8 × 8	0.4
4	12 × 12 × 12	0.1
5	12 × 12 × 12	0.2
6	12 × 12 × 12	0.4
7	20 × 20 × 20	0.1
8	20 × 20 × 20	0.2
9	20 × 20 × 20	0.4

**Table 2:** Analysis of variance for PC.

Source	DF	Adj SS	Adj MS	F-Value	*p*-Value
Specimen size	2	0.004207	0.002104	0.62	0.545
layer height	2	0.096618	0.048309	14.19	0.000
Specimen size × layer height	4	0.002783	0.000696	0.20	0.934
Error	33	0.112318	0.003404		
Total	41	0.215120			

**Table 3:** Analysis of variance for TPU.

Source	DF	Adj SS	Adj MS	F-Value	*p*-Value
Specimen size	2	0.000418	0.000209	99.03	0.000
layer height	2	0.000083	0.000041	19.60	0.000
Specimen size × layer height	4	0.000086	0.000022	10.22	0.000
Error	27	0.000057	0.000002		
Total	35	0.000644			

## Data Availability

The original contributions presented in the study are included in the article, further inquiries can be directed to the corresponding authors.

## Appendix A

The compression moduli for TPU and PC are reported in this section.

**Table A1:** Compression modulus for TPU.

Cube Dimensions (mm^3^)	Layer Height (mm)	Compression Modulus (GPa)	Standard Deviation
20 × 20 × 20	0.1	0.025	0.001
20 × 20 × 20	0.2	0.025	0.001
20 × 20 × 20	0.4	0.027	0.001
12 × 12 × 12	0.1	0.028	0.002
12 × 12 × 12	0.2	0.032	0.001
12 × 12 × 12	0.4	0.030	0.002
8 × 8 × 8	0.1	0.031	0.004
8 × 8 × 8	0.2	0.038	0.001
8 × 8 × 8	0.4	0.032	0.001

**Table A2:** Compression modulus for PC.

Cube Dimensions (mm^3^)	Layer Height (mm)	Compression Modulus (GPa)	Standard Deviation
20 × 20 × 20	0.1	0.886	0.048
20 × 20 × 20	0.2	0.907	0.027
20 × 20 × 20	0.4	0.819	0.050
12 × 12 × 12	0.1	0.884	0.038
12 × 12 × 12	0.2	0.911	0.028
12 × 12 × 12	0.4	0.788	0.033
8 × 8 × 8	0.1	0.873	0.042
8 × 8 × 8	0.2	0.894	0.083
8 × 8 × 8	0.4	0.769	0.092

## Appendix B

Analysis of variance (ANOVA) is a statistical test that evaluates the difference between the means of two or more groups. It uses the following hypothesis testing to determine if the factors under consideration can produce a statistical difference in the mean of the measured quantity.

ANOVA hypothesis:

Null hypothesis: All means are equal
Alternative hypothesis: Not all means are equal

Anderson–Darling test: One of the prerequisites for ANOVA is that the response obtained from the experiments should be normally distributed. The Anderson–Darling test is used to determine if the assumption is valid. It is a statistical method that assesses how well the data follow a given distribution.

The hypotheses for the Anderson–Darling test are:

Null hypothesis: The data follow a specified distribution
Alternative hypothesis: The data do not follow a specified distribution

ANOVA can be performed once it has been determined that all the assumptions required for ANOVA are satisfied. A simplified one-way ANOVA can be performed as follows:

Assuming *k* factors and *n* number of observations for each factor, the number of observations can be arranged as follows:

$$y=\left[\begin{array}{cccc} y_{11} & y_{12} & \cdots & y_{1k}\\ y_{21} & y_{22} & \cdots & y_{2k}\\ \vdots & \vdots & \ddots & \vdots \\ y_{n1} & y_{n2} & \cdots & y_{nk} \end{array}\right]$$

where $y_{ij}$ represents the $i^{th}$ observation corresponding to factor *j*.

ANOVA examines the difference in the group means by partitioning the total variation in the data into two ways:

- Variation in group means from the overall mean: $\bar{y_{j}}-\bar{y_{..}}$, where $\bar{y_{j}}$ is the group mean and $\bar{y}$ is the overall mean.
- Variation within the group: $y_{ij}-\bar{y_{j}}$

Using the above variations, the total sum of squares (*SST*) can be partitioned into a sum of squares due to the between-groups effect (*SSR*) and the sum of squared errors (*SSE*). Mathematically, it is given by

(A1) SST=SSR+SSE

(A2) $$\sum_{i}\sum_{j}{\left(y_{ij}-\bar{y_{..}}\right)}^{2}=\sum_{j}n_{j}{\left(\bar{y_{j}}-\bar{y_{..}}\right)}^{2}+\sum_{i}\sum_{j}{\left(y_{ij}-\bar{y_{j}}\right)}^{2}$$

Similarly, the total degrees of freedom is the sum of the degrees of freedom (*DF*) of factors and errors:

(A3) $$\begin{array}{c} k\cdot n-1=(k-1)+k\cdot (\alpha -1)\\ DF_{T}=DF_{Level}+DF_{E} \end{array}$$

The value *F* (see the description of *F* below) can be calculated as follows:

(A4) $$F=\frac{SSR/(k-1)}{SSE/(N-k)}=\frac{MSR}{MSE}$$

where MSR is the mean squared treatment, MSE is the mean squared error, and *N* is the total number of observations.

Based on the above calculations, the terms in Table 2 and Table 3 can be defined as follows:

- *DF*: Stands for the degrees of freedom. It assesses the number of independent pieces of information in the provided data.
- Adj SS: The SS stands for the sum of squares, which represents the deviation from the mean. It is calculated as a summation of the squares of the differences from the mean. In adjusted sums of squares, the order in which factors are rendered does not affect the values.
- Adj MS: It is calculated by dividing the sum of squares by the corresponding degree of freedom.
- *F*-value: The ratio between two variances. It is used to determine the ratio of explained variance to unexplained variance. Along with the *p*-value, it is used to determine if the results are significant enough to reject the null hypothesis. A sufficiently large F-value indicates that the term or model is significant.
- *p*-value: This is a measure of probability that assesses the level of evidence against the null hypothesis. A lower *p*-value indicates a stronger rejection of the null hypothesis.

## References

[1] Ramakrishna D., Bala Murali G. (2022). Bio-inspired 3D-printed lattice structures for energy absorption applications: A review. Proc. Inst. Mech. Eng. Part L J. Mater. Des. Appl..

[2] Wei J., Pan F., Ping H., Yang K., Wang Y., Wang Q., Fu Z. (2023). Bioinspired Additive Manufacturing of Hierarchical Materials: From Biostructures to Functions. Research.

[3] Du Y., Gu D., Xi L., Dai D., Gao T., Zhu J., Ma C. (2020). Laser additive manufacturing of bio-inspired lattice structure: Forming quality, microstructure and energy absorption behavior. Mater. Sci. Eng. A.

[4] He J., Kushwaha S., Mahrous M.A., Abueidda D., Faierson E., Jasiuk I. (2023). Size-dependence of AM Ti–6Al–4V: Experimental characterization and applications in thin-walled structures simulations. Thin-Walled Struct..

[5] Roach A.M., White B.C., Garland A., Jared B.H., Carroll J.D., Boyce B.L. (2020). Size-dependent stochastic tensile properties in additively manufactured 316L stainless steel. Addit. Manuf..

[6] Cui H., Hensleigh R., Chen H., Zheng X. (2018). Additive Manufacturing and size-dependent mechanical properties of three-dimensional microarchitected, high-temperature ceramic metamaterials. J. Mater. Res..

[7] Jia H., Sun H., Wang H., Wu Y., Wang H. (2022). Size effect in selective laser melting additive manufacturing of 700 mm large component. J. Manuf. Process..

[8] Fotovvati B., Asadi E. (2019). Size effects on geometrical accuracy for additive manufacturing of Ti-6Al-4V ELI parts. Int. J. Adv. Manuf. Technol..

[9] Srivatsan T., Sudarshan T. (2015). Additive Manufacturing: Innovations, Advances, and Applications.

[10] Chadha C., James K., Jasiuk I.M., Patterson A.E. (2022). Extending the operating life of thermoplastic components via on-demand patching and repair using fused filament fabrication. J. Manuf. Mater. Process..

[11] Ceruti A., Marzocca P., Liverani A., Bil C. (2019). Maintenance in aeronautics in an Industry 4.0 context: The role of Augmented Reality and Additive Manufacturing. J. Comput. Des. Eng..

[12] Haryńska A., Carayon I., Kosmela P., Szeliski K., Łapiński M., Pokrywczyńska M., Kucińska-Lipka J., Janik H. (2020). A comprehensive evaluation of flexible FDM/FFF 3D printing filament as a potential material in medical application. Eur. Polym. J..

[13] Volpe S., Petrella A., Sangiorgio V., Notarnicola M., Fiorito F. (2021). Preparation and characterization of novel environmentally sustainable mortars based on magnesium potassium phosphate cement for additive manufacturing. AIMS Mater. Sci..

[14] Nurizada A., Kirane K. (2020). Induced anisotropy in the fracturing behavior of 3D printed parts analyzed by the size effect method. Eng. Fract. Mech..

[15] Sadaghian H., Dadmand B., Pourbaba M., Jabbari S., Yeon J.H. (2023). The Effect of Size on the Mechanical Properties of 3D-Printed Polymers. Sustainability.

[16] Wu C., Chen C., Cheeseman C. (2021). Size effects on the mechanical properties of 3D printed plaster and PLA parts. J. Mater. Civ. Eng..

[17] Zhang G., Wang Q., Ni Y., Liu P., Liu F., Leguillon D., Xu L.R. (2023). A systematic investigation on the minimum tensile strengths and size effects of 3D printing polymers. Polym. Test..

[18] Bažant Z.P., Kazemi M.T. (1990). Size Effect in Fracture of Ceramics and Its Use To Determine Fracture Energy and Effective Process Zone Length. J. Am. Ceram. Soc..

[19] Bazant Z.P., Chen E.P. (1997). Scaling of structural failure. Appl. Mech..

[20] Carpinteri A., Chiaia B. (1997). Multifractal scaling laws in the breaking behaviour of disordered materials. Chaos Solitons Fractals.

[21] Bell D., Siegmund T. (2018). 3D-printed polymers exhibit a strength size effect. Addit. Manuf..

[22] Guessasma S., Belhabib S., Nouri H., Ben Hassana O. (2016). Anisotropic damage inferred to 3D printed polymers using fused deposition modelling and subject to severe compression. Eur. Polym. J..

[23] Guessasma S., Belhabib S., Nouri H. (2015). Significance of pore percolation to drive anisotropic effects of 3D printed polymers revealed with X-ray/*mu*-tomography and finite element computation. Polymer.

[24] Patterson A.E., Chadha C., Jasiuk I.M. (2022). Manufacturing process-driven structured materials (MPDSMs): Design and fabrication for extrusion-based additive manufacturing. Rapid Prototyp. J..

[25] Li B., Zhao M., Wan X. The influence of void distribution on transverse mechanical properties of unidirectional composites. Proceedings of the 2017 8th International Conference on Mechanical and Aerospace Engineering (ICMAE).

[26] Paux J., Ginoux G., Pulickan S., Allaoui S. (2023). Influence of printing irregularities on the elastic behavior and mesostructural stress concentrations in material extrusion additive manufacturing—A numerical approach based on X-ray tomography. Addit. Manuf..

[27] Lei M., Wang Y., Wei Q., Li M., Zhang J., Wang Y. (2023). Micromechanical modeling and numerical homogenization calculation of effective stiffness of 3D printing PLA/CF composites. J. Manuf. Process..

[28] Sheikh T., Behdinan K. (2023). Geometric void-multiscale model for evaluating the effect of bead width and layer height on voids in FDM parts. Rapid Prototyp. J..

[29] Tagscherer N., Schromm T., Drechsler K. (2022). Foundational Investigation on the Characterization of Porosity and Fiber Orientation Using XCT in Large-Scale Extrusion Additive Manufacturing. Materials.

[30] Hernandez-Contreras A., Ruiz-Huerta L., Caballero-Ruiz A., Moock V., Siller H.R. (2020). Extended CT Void Analysis in FDM Additive Manufacturing Components. Materials.

[31] Rajpurohit S.R., Dave H.K. (2018). Effect of process parameters on tensile strength of FDM printed PLA part. Rapid Prototyp. J..

[32] Fang L., Yan Y., Agarwal O., Seppala J.E., Hemker K.J., Kang S.H. (2020). Processing-structure-property relationships of bisphenol-A-polycarbonate samples prepared by fused filament fabrication. Addit. Manuf..

[33] Biswas P., Guessasma S., Li J. (2019). Numerical prediction of orthotropic elastic properties of 3D-printed materials using micro-CT and representative volume element. Acta Mech..

[34] Garzon-Hernandez S., Garcia-Gonzalez D., Jérusalem A., Arias A. (2020). Design of FDM 3D printed polymers: An experimental-modelling methodology for the prediction of mechanical properties. Mater. Des..

[35] Ulkir O., Ertugrul I., Ersoy S., Yağımlı B. (2024). The Effects of Printing Temperature on the Mechanical Properties of 3D-Printed Acrylonitrile Butadiene Styrene. Appl. Sci..

[36] Kechagias J.D., Vidakis N., Petousis M. (2021). Parameter effects and process modeling of FFF-TPU mechanical response. Mater. Manuf. Process..

[37] Kechagias J., Zaoutsos S. (2024). Effects of 3D-printing processing parameters on FFF parts’ porosity: Outlook and trends. Mater. Manuf. Process..

[38] Bellehumeur C., Li L., Sun Q., Gu P. (2004). Modeling of bond formation between polymer filaments in the fused deposition modeling process. J. Manuf. Process..

[39] Yao T., Ouyang H., Dai S., Deng Z., Zhang K. (2021). Effects of manufacturing micro-structure on vibration of FFF 3D printing plates: Material characterisation, numerical analysis and experimental study. Compos. Struct..

[40] Nouri H., Guessasma S., Belhabib S. (2016). Structural imperfections in additive manufacturing perceived from the X-ray micro-tomography perspective. J. Mater. Process. Technol..

[41] Zouaoui M., Gardan J., Lafon P., Makke A., Labergere C., Recho N. (2021). A Finite Element Method to Predict the Mechanical Behavior of a Pre-Structured Material Manufactured by Fused Filament Fabrication in 3D Printing. Appl. Sci..

[42] Wang X., Zhao L., Fuh J.Y.H., Lee H.P. (2019). Effect of Porosity on Mechanical Properties of 3D Printed Polymers: Experiments and Micromechanical Modeling Based on X-Ray Computed Tomography Analysis. Polymers.

[43] Polyzos E., Hemelrijck D.V., Pyl L. (2022). Influence of void contour on the elastic behavior of parts produced by material extrusion. Addit. Manuf..

[44] Colón Quintana J.L., Osswald T. (2022). Understanding softening of amorphous materials for FFF applications. Int. Polym. Process..

[45] Gorbunova M.A., Anokhin D.V., Abukaev A.F., Ivanov D.A. (2023). The Influence of Long-Time Storage on the Structure and Properties of Multi-Block Thermoplastic Polyurethanes Based on Poly(butylene adipate) Diol and Polycaprolactone Diol. Materials.

[46] Vaes D., Van Puyvelde P. (2021). Semi-crystalline feedstock for filament-based 3D printing of polymers. Prog. Polym. Sci..

[47] Mahrous M.A., Chadha C., Robins P.L., Bonney C., Boateng K.A., Meyers M., Jasiuk I. (2023). Multimodule imaging of the hierarchical equine hoof wall porosity and structure. J. Mater. Res. Technol..

[48] (2023). Standard Test Method for Compressive Properties of Rigid Plastics.

[49] Eiliat H., Urbanic R.J. (2018). Minimizing voids for a material extrusion-based process. Rapid Prototyp. J..

[50] Lin X., Gao J., Wang J., Wang R., Gong M., Zhang L., Lu Y., Wang D., Zhang L. (2021). Desktop printing of 3D thermoplastic polyurethane parts with enhanced mechanical performance using filaments with varying stiffness. Addit. Manuf..

[51] Kasmi S., Ginoux G., Labbé E., Alix S. (2021). Multi-physics properties of thermoplastic polyurethane at various fused filament fabrication parameters. Rapid Prototyp. J..

[52] Hebda M., McIlroy C., Whiteside B., Caton-Rose F., Coates P. (2019). A method for predicting geometric characteristics of polymer deposition during fused-filament-fabrication. Addit. Manuf..

[53] Lin X., Coates P., Hebda M., Wang R., Lu Y., Zhang L. (2020). Experimental analysis of the tensile property of FFF-printed elastomers. Polym. Test..

[54] Coogan T.J., Kazmer D.O. (2020). Prediction of interlayer strength in material extrusion additive manufacturing. Addit. Manuf..

[55] Seppala J.E., Hoon Han S., Hillgartner K.E., Davis C.S., Migler K.B. (2017). Weld formation during material extrusion additive manufacturing. Soft Matter.

[56] Bhalodi D., Zalavadiya K., Gurrala P.K. (2019). Influence of temperature on polymer parts manufactured by fused deposition modeling process. J. Braz. Soc. Mech. Sci. Eng..

[57] Polychronopoulos N.D., Vlachopoulos J. (2020). The role of heating and cooling in viscous sintering of pairs of spheres and pairs of cylinders. Rapid Prototyp. J..

